# Failure rates of common grafts used in ACL reconstructions: a systematic review of studies published in the last decade

**DOI:** 10.1007/s00402-021-04147-w

**Published:** 2021-09-18

**Authors:** Gerwin Haybäck, Christoph Raas, Ralf Rosenberger

**Affiliations:** 1 Universitätsklinik für Orthopädie und Traumatologie, Landeskrankenhaus Salzburg, Paracelsus Medizinische Privatuniversität, Müllner Hauptstraße 48, 5020 Salzburg, Austria; 2 PRIVATKLINIK HOCHRUM, Sanatorium der Kreuzschwestern, Lärchenstraße 41, 6063 Rum, Austria

**Keywords:** ACL reconstruction, Graft failure rate, Autograft, Allograft, Quadriceps, Bone–patellar tendon–bone, Hamstring

## Abstract

**Introduction:**

In this review paper, graft failure rates of different graft types (hamstring tendon autografts, bone–patellar tendon–bone autografts, quadriceps tendon autografts and diverse allografts) that are used for surgical reconstruction of the anterior cruciate ligament are compared and statistically analysed.

**Methods:**

Literature search was conducted in PubMed according to Preferred Reporting Items for Systematic Reviews and Meta-Analysis (PRISMA) criteria. A total of 194 studies, which reported graft failure rates of at least one of the anterior cruciate ligament reconstruction methods mentioned above, were included in this systematic review. To be able to compare studies with different follow-up periods, a yearly graft failure rate for each reconstruction group was calculated and then investigated for significant differences by using the Kruskal–Wallis test.

**Results:**

Overall, a total of 152,548 patients treated with an anterior cruciate ligament reconstruction were included in the calculations. Comparison of graft types showed that hamstring tendon autografts had a yearly graft failure rate of 1.70%, whereas the bone–patellar tendon–bone autograft group had 1.16%, the quadriceps tendon autograft group 0.72%, and the allografts 1.76%.

**Conclusion:**

The findings of this meta-data study indicate that reconstructing the anterior cruciate ligament using quadriceps tendon autografts, hamstring tendon autografts, patellar tendon autografts or allografts does not show significant differences in terms of graft failure rates.

**Supplementary Information:**

The online version contains supplementary material available at 10.1007/s00402-021-04147-w.

## Introduction

Isolated tear of the anterior cruciate ligament (ACL) is a common orthopaedic injury, as the annual incidence of 68.6 per 100,000 person-years of the US population shows [1]. Although treatment options for a torn ACL are discussed controversially, superior outcomes of anterior cruciate ligament reconstruction (ACLR) compared to non-surgical treatment are reported in terms of quality of life and function in sports [2]. Recent findings in a systematic review of Krause et al. suggest that there is a tendency to better functional outcome and knee stability when performing ACLR compared to conservative treatment [3].

Several factors can influence the outcome of an ACLR and should therefore be taken into consideration when graft survival is investigated. Age has been identified as a risk factor for ACL graft rupture. The odds of an ACL graft rupture decreases with every yearly increase in patient age [4]. However, sex is not considered as a risk factor of increased ACLR failure rate or clinically important difference in patient-reported outcomes [5].

In recent traumatology surgery, several ways of reconstructing a ruptured ACL were established. In a global perspective, the most common grafts used for reconstruction of the ACL are hamstring tendon autografts (HTA), bone–patellar tendon–bone autografts (BPTBA), different allografts and quadriceps tendon autografts (QTA), respectively. As a matter of fact, single-bundle reconstruction is used more frequently than double-bundle technique. [6]

The purpose of this systematic review was to compare graft failure rates of different graft options used for ACLR by taking the most recent studies with a very high volume of patients into calculation. Such an approach to this topic has not yet been chosen in recent literature. A recent review paper including far less trials than our systematic review suggested that there is no significant difference between autograft subgroups in terms of graft failure rates [7]. Between autografts and allografts, there might be no significant long-term difference in failure rates as well [8, 9]. Consequently, the main hypothesis stating that there is no statistically significant difference in graft failure rates within the autograft subgroups and between autograft subgroups and allografts was defined. The results of the present study may contribute to a better understanding and orientation in terms of graft choice and functional survival of different graft types.

## Materials and methods

### Literature search

A systematic literature search on common methods for ACLR using autografts and allografts was conducted in PubMed based on the Preferred Reporting Items for Systematic Reviews and Meta-Analyses (PRISMA) criteria. Search was performed throughout May 2020 using the following terms for search: “ACL reconstruction” in combination with “quadriceps tendon” OR “quadriceps graft” OR “hamstring” OR “bone tendon bone” OR “patellar tendon” OR “allograft” in <All Fields> .

### Eligibility and study selection

All obtained papers’ titles and abstracts were screened, and relevant studies were identified. To further assess these studies for eligibility, certain selection criteria were defined.

Inclusion criteria:

- Publication date within the last 10 years (May 2010–May 2020).
- Trials only conducted on human species (level of evidence I-IV).
- A follow-up (FU) period of at least 2 years.
- Isolated ACL rupture as primary ligamentous injury with or without meniscal damage.
- Study population with a minimum mean age of 18 years.
- All tibial and femoral graft fixation techniques.
- Studies including transtibial or anteromedial tibial tunnel drilling.
- All kinds of hamstring techniques (any number of strands).
- All kinds of sterilisation and preservation methods for allografts.

Exclusion criteria:

- Studies published in languages other than English.
- No documentation of graft failure or re-rupture rate.
- Revision ACLR.
- Studies investigating only highest-risk populations such as professional athletes or skeletally immature.
- All double bundle or hybrid graft techniques.
- Multiple ligamentous injuries of the knee joint.
- Follow-up duration unavailable or only available as median.

**Fig. 1:**
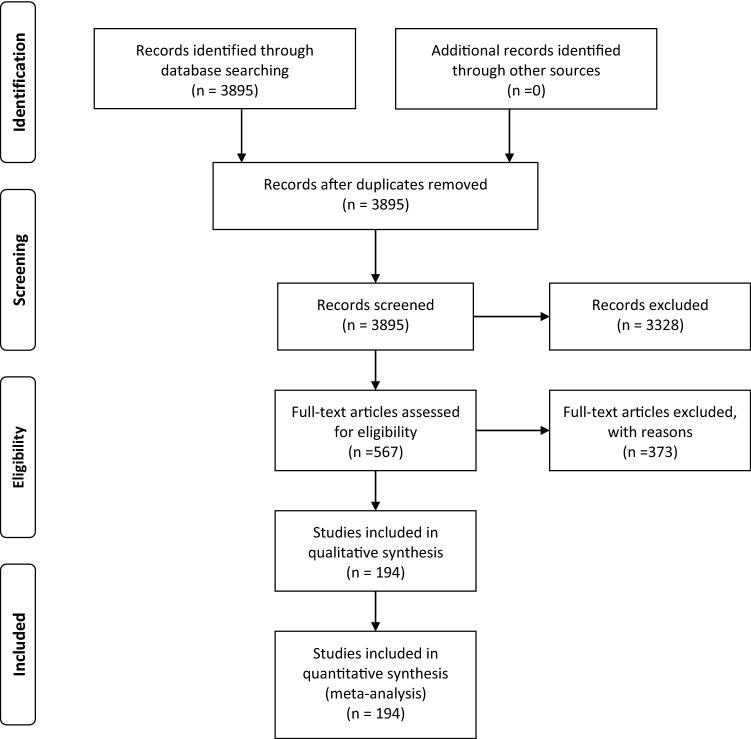
PRISMA flow diagram

A total of 3895 citations were found, and 194 studies were selected to be eligible for the systematic review according to the inclusion and exclusion criteria, as Fig. 1 shows.

### Data extraction

To create homogenous data about study characteristics and patient demographics, information was collected by systematically extracting year of publication, authors, study design and level of evidence as well as number of subjects, mean age, mean follow-up time, cases of graft failure and used grafts from the studies.

From this dataset, the main patient demographics containing number of operated patients, the mean age and mean follow-up time were elaborated. These main patient demographics are displayed in Table 1.

**Table 1:** Patient demographics

Graft type	Patients (*n*)	Mean age (years)	Mean FU (months)
HTA	97,385	28.5	53.3
BPTBA	37,524	27.7	86.9
QTA	1222	30.2	36.7
Allograft	16,417	30.5	47.5
Total	152,548	28.7	61.5

*HTA* hamstring tendon autograft, *BPTBA* bone–patellar tendon bone autograft, *QTA* quadriceps tendon autograft

### Methodological quality assessment

The methodological index for non-randomized studies (MINORS) was used to assess methodological quality of both non-randomized comparative studies and non-randomized non-comparative studies that were included in this systematic review [10]. The MINORS instrument consists of an 8-item index (global ideal score of 16) for non-comparative studies and a 12-item index (global ideal score of 24) for comparative studies. As Carl et al. suggested, studies scoring more than 12 out of 16 points or more than 19 out of 24 points were considered high quality [11].

The quality of the included randomized controlled trials was assessed using the modified Jadad scale [12]. The overall score for each study ranged from 0 to 8 points. Randomized controlled trials were considered to be of high quality if they reached 4–8 points and of low quality if they achieved 0–3 points as suggested elsewhere [13].

### Statistical analysis

For statistical calculation of the results, IBM SPSS Statistics 23 (IBM Corporation, Armonk, NY, USA) was used. At first, descriptive statistics were carried out: the number of patients included, the average patients’ age at surgery and overall mean follow-up was calculated per graft type and for the overall study population.

The follow-up duration of the 194 studies, which were included in this systematic review, ranged from 2 years up to 25 years. A yearly graft failure rate (percentage) was calculated for each graft type to make studies with different follow-up periods comparable. This variable (yearly graft failure rate) was then examined for normal distribution using the Kolmogorov–Smirnov test. To examine if the grafts differ in yearly graft failure rates, the Kruskal–Wallis test was conducted. In this testing method, the null hypothesis assumed no significant difference in yearly failure rates between the reconstruction groups. The level of significance was set at a *p* value of less than 0.05.

## Results

194 studies of different study types and different levels of evidence (I–IV) were included in this systematic review. Some of the studies included more than one of the four graft types, resulting in 296 cohorts in the dataset, each cohort containing information about one of the reconstruction methods. Of these 296 cohorts, the distribution to graft types was 148 (50.0%), 87 (29.4%), 12 (4.1%) and 49 (16.5%) for hamstring autograft, bone–patellar tendon–bone autograft, quadriceps autograft and allografts, respectively (Table 2).

**Table 2:** Distribution of study cohorts to reconstruction methods

Graft type	Study cohorts (n)	Percent (%)
HTA	148	50.0
BPTBA	87	29.4
QTA	12	4.1
Allograft	49	16.5
Total	296	100.0

*HTA* hamstring tendon autograft, *BPTBA* bone–patellar tendon bone autograft, *QTA* quadriceps tendon autograft

### Quality of the included studies

The methodological quality of non-randomized studies was assessed according to the MINORS criteria. The average score for non-randomized studies was 77% of maximum (maximum 18 or 24 points, depending on whether the study design was comparative or not). 173 studies were assessed this way. 81 of them were regarded as high-quality studies.

The included randomized controlled trials (assessed with the modified Jadad scale) reached an average of 6.4 out of 8 points (80% of maximum). All of the 21 assessed randomized controlled trials were regarded as high-quality trials. Detailed tabular results of the methodological quality assessment, study type and level of evidence of the included studies can be found in the appendix.

### Comparison of graft failure rates of all graft types

The Kruskal–Wallis test of yearly failure rates for the four main graft groups showed a *p* value of 0.072 which is considered as not significant (*p *> 0.05). Thus, the yearly graft failure rate of the four investigated ACLR groups did not differ significantly and the null hypothesis was retained.

The mean of the yearly failure rate was calculated for each graft type and is displayed in Table 3. The QTA group had the lowest mean yearly graft failure rate (0.72%), followed by the BPTBA group (1.16%), the HTA group (1.70%) and the allograft group (1.76%).

**Table 3:** Yearly graft failure rates of all graft types

Graft type	Mean (%)	Std. deviation	Median	Range
HTA	1.70	1.98	1.07	11.11
BPTBA	1.16	1.38	0.76	8.96
QTA	0.72	0.88	0.33	2.35
Allograft	1.76	2.41	1.26	14.58
Total	1.51	1.89	0.99	14.58

*HTA* hamstring tendon autograft, *BPTBA* bone–patellar tendon–bone autograft, *QTA* quadriceps tendon autograft

In terms of follow-up time, the BPTB autograft group had an average of 86.9 months follow-up duration, the hamstring autograft group 53.3 months, the allograft group 47.5 and the QTA group was followed up with a mean of 36.7 months (Table 1).

## Discussion

The most important finding of this systematic review is that all types of grafts that are commonly used for ACLR did not differ significantly in terms of yearly graft failure rates. These main findings confirm the results of a recent meta-analysis by Mouarbes et al. in which HTA, BPTBA and QTA were compared and no significant difference in graft failure rates could be found [7].

Recent findings of Nyland et al. displayed that QTA produces better results in terms of graft failure rates than other common grafts [14]. However, this tendency could not be confirmed by the most recent studies by Lind et al. and Galan et al. as well as this systematic review. Lind et al. focussed on comparing graft failure rates of the QTA to other options and reported a revision rate of 4.7% out of 531 cases for QTA in a 2-year-follow-up period. This failure rate was significantly higher than the failure rates of hamstring and patellar tendon grafts. Galan and colleagues conducted a 5-year follow-up study. The failure rate for QTA was 10.7% (31) out of 291 cases. These recent findings can be a hint that failure rates of QTA may increase disproportionately with longer follow-up duration. Due to the fact that frequent use of QTA in ACL reconstruction surgery was pushed in the past few years, there is a lack of long-term follow-up studies investigating this graft option. The scientific debate about ACL reconstruction surgery could benefit from high-quality studies investigating long-term (10 years and more) failure rates and functional outcomes of the QTA [15, 16].

Furthermore, a lot of quality research has been done on the comparison of graft failure rates of HTA with BPTBA in the last decade [17]. Graft failure rates of HTA showed no significant difference when compared to BPTBA [18]. These findings also match the results of this systematic review.

Graft failure may be the most important indicator for measuring success of an ACLR, but there are still other considerable factors such as harvest site pain and functional results. A well-known downside of BPTBA is that anterior knee pain and kneeling pain often occur due to the harvest site defect [19]. This essential aspect prevents that BPTBA is regarded superior to HTA, although in some studies failure rates were lower and functional outcomes were better [17, 20, 21].

In terms of functional outcomes and harvest site morbidity, the QTA is regarded as a promising alternative for ACLR compared to current gold standard methods (HTA and BPTBA). Slone et al. stated that stability outcomes (Lachman, pivot-shift and instrumented laxity testing), functional outcomes (International Knee Documentation Committee and Lysholm scores), range of motion, overall patient satisfaction and complications were similar when comparing quadriceps tendon autograft to other graft options [22–24]. Contrarily to that, Nyland and colleagues found significantly better results in pivot shift laxity for QTA than for HTA [14]. In terms of donor site morbidity or harvest site pain, ACLR using autologous quadriceps tendon achieved better results than BPTBA and HTA [7, 22].

In addition, graft selection should always be done thoroughly considering the patient’s age, needs, activity level and concomitant injuries. By implication, having many valid graft options available is advantageous and surely does enrich the quality of ACL surgery as all of them deliver predominantly satisfying results.

The promising results achieved by quadriceps tendon autograft inevitably lead to the implication that the quadriceps tendon could be used for allograft ACLR as well. Up to now, hardly any high-quality studies including the QT allografts have been carried out. Kwak and colleagues published a matched case control study comparing Quadriceps tendon *autograft* with Quadriceps tendon *allografts* [25]. This study did not reveal any statistically significant differences between the two groups in terms of functional outcomes, complications and re-ruptures. These findings may be a hint that the quadriceps tendon can also achieve good outcomes in ACLR when used as allograft. Thus, doing more research on this graft option could possibly result in highly relevant discussions further improving ACL reconstruction surgery.

There are several limitations to this study. Firstly, different study types of different levels of evidence (I-IV) were included and equally weighted in this review. Overall, 102 of 194 included studies were high-quality studies. Secondly, the studies that were included in the present systematic review contained different graft fixation methods such as suspensory fixation methods, fixation with interference screws or a combination of both methods. Thirdly, the cohort size of the included studies varied from 8 to 17.096 patients (e.g. nationwide cohort studies). Furthermore, the definition of graft failure was inconsistent as some studies defined the need for ACL revision as graft failure, while others considered pathological magnetic resonance imaging (MRI) or clinical deficits as graft failure. Another important aspect that should be kept in mind in this respect is the question whether traumatic re-rupture of the reconstructed ACL should be considered as graft failure or re-injury [26]. As a final consensus could not be achieved in literature, several studies using slightly different definitions of graft failure were included in this systematic review.

As a matter of fact, this systematic review has certain strengths. To our knowledge, this is by far the largest systematic review paper: a great number of studies (194) and ACL reconstructions (more than 150,000) were included. Only studies published in the past 10 years were selected for this review in order to ensure actuality of surgical procedures to a certain extent. By calculating a yearly graft failure rate, the results of 194 different clinical studies were combined and compared in an effective and comprehensible way.

## Conclusion

The findings of this extensive systematic review showed no significant differences in yearly graft failure rates of HTA, BPTBA, QTA and allografts. Based on these data, all these graft options deliver comparable results in terms of graft failure rates and therefore every graft type could be rightly considered as reliable option for ACLR. An increased use of QTA that will ultimately be documented in publications of more long-term studies will show whether a positive or negative impact on failure rates of this graft can be detected.

## Supplementary Information

Below is the link to the electronic supplementary material.

Supplementary file1 (XLSX 32 KB)

Supplementary file2 (SAV 14 KB)

## Abbreviations

ACL: Anterior cruciate ligament
ACLR: Anterior cruciate ligament reconstruction
HTA: Hamstring tendon autograft
BPTBA: Bone–patellar tendon–bone autograft
QT(A): Quadriceps tendon (autograft)
PRISMA: Preferred Reporting Items for Systematic Reviews and Meta-Analysis
MINORS: Methodological index for non-randomized studies
FU: Follow-up
MRI: Magnetic resonance imaging
